# Streamlining urology cancer multidisciplinary team meetings: Implementation and outcomes

**DOI:** 10.1002/bco2.70167

**Published:** 2026-02-05

**Authors:** Hossein Arang, Adeyinka Pratt, Muhammed Rashim Parappan, Mutahhar Nabeel Syed, Jayasimha Abbaraju, Srijit Banerjee, Sanjeev Madaan

**Affiliations:** ^1^ Department of Urology & Nephrology Dartford and Gravesham NHS Trust, Darent Valley Hospital Kent UK; ^2^ Faculty of Medicine, Health & Social Care Canterbury Christ Church University Kent UK

**Keywords:** MDT streamlining, multidisciplinary team, NHS cancer services, service evaluation, Standards of Care, urology cancer

## Abstract

**Objectives:**

This work aimed to evaluate the implementation and impact of a pre‐multidisciplinary team (MDT) triage model using Standards of Care (SoC) for streamlining in a urology cancer service.

**Materials and Methods:**

An SoC framework was developed by the cancer lead in line with national guidance. Using this, the Urology MDT at Dartford and Gravesham NHS Trust (DGT) introduced a pre‐MDT triage model. Each week, a consultant urologist, clinical nurse specialist, and MDT coordinator reviewed referrals and allocated them for either full MDT discussion or protocolised management. Data were collected over 50 weeks, including meeting duration, case numbers, plan changes, and a clinician survey.

**Results:**

Average MDT duration reduced from 158 to 135 min (*p* < 0.001), allowing more focused discussion of complex cases. Of 50 MDT members invited, 25 responded to the survey (50%): 79% were confident that SoC‐aligned cases received appropriate management without full discussion, 84% supported ongoing streamlining, and most reported time savings. Suggested improvements included artificial intelligence (AI) decision‐support and clearer timelines. Overall, streamlining improved both the operational efficiency and clinician satisfaction.

**Conclusion:**

The initiative enhanced MDT efficiency, maintained oversight, and strengthened clinician confidence in protocolised care pathways. Challenges remain, including reliance on a single consultant for triage and occasional gaps in the availability of radiology or histopathology reports, which may affect sustainability. Overall, these findings support wider adoption of pre‐MDT streamlining in cancer services.

## 1 INTRODUCTION

Cancer care depends on coordinated input from multidisciplinary teams (MDTs) to ensure management is both evidence‐based and patient‐centred. The Multidisciplinary Team Meeting model (MDTM) has become the established structure for collective decision‐making, with weekly meetings bringing together surgeons, oncologists, radiologists, pathologists, and specialist nurses under the direction of a lead clinician.^1^, ^2^

In recent years, however, the workload of MDTs has increased sharply. An ageing population, more sensitive diagnostic pathways, and expanding therapeutic options have added both volume and complexity to the cases presented. As a result, meetings have lengthened, while the time available for each case has narrowed. Important aspects such as comorbidities, patient preferences, and eligibility for clinical trials are sometimes incompletely addressed.^3^, ^4^

To relieve this pressure, NHS England issued the *Streamlining Multi‐Disciplinary Team Meetings Guidance for Cancer Alliances*.^5^ The guidance sets out a framework for case stratification alongside the application of Standards of Care (SoCs). SoCs define specific points in the patient pathway at which guideline‐recommended interventions can be applied without the need for full team discussion.

When applied consistently, SoCs allow routine cases to be processed efficiently while still under MDT oversight. This preserves meeting capacity for complex presentations, supports more robust decision‐making, and promotes the systematic recording of essential clinical information such as comorbidity, performance status, and patient preference. These priorities align closely with the NHS Long Term Plan.^5^, ^6^

This study examines the implementation of MDTM streamlining within the SoC framework by the Urology service at Dartford and Gravesham NHS Trust (DGT).

## 2 MATERIALS AND METHODS

This work was conducted as a service evaluation of MDT streamlining in routine clinical practice and was undertaken under local cancer alliance and Integrated Care Board (ICB) governance and followed the NHS England guidance on this topic. The DGT Urology team launched a pre‐MDT triage model as part of broader efforts to improve MDT efficiency. The cancer lead initiated the project and appointed a project manager to support delivery under appropriate clinical oversight. The model was designed to reduce the number of cases requiring complete MDT discussion by identifying those suitable for protocolised management through pre‐review.

An initial SoC framework was developed by the cancer lead and subsequently circulated among all members of the MDT for their input. Following feedback, the SoC was finalised and formally approved. Simultaneously, consensus was achieved regarding the pre‐MDT team, consisting of a single consultant urologist, a cancer nurse specialist, and the MDT coordinator. The team created SoC pathways for prostate, bladder, renal cell carcinoma, upper tract urothelial carcinoma, testicular, and penile cancers. These pathways provided structured risk stratification and played a central role in achieving efficiency gains during the pilot. The streamlining pilot was implemented in January 2024. Early on, some clinicians expressed concern that streamlining could undermine multidisciplinary input and compromise quality of decisions. To address this, the team clarified that all cases, streamlined or not, would remain on the main MDT list. Any MDT member could challenge a pre‐MDT decision based on pathology, imaging, comorbidities, or specialist input.

To enable thorough and timely case preparation, dedicated protected time is allocated within the consultant urologist's job plan specifically for pre‐MDT review. This protected session allows a comprehensive assessment of each patient's clinical information, including imaging, pathology, and electronic health records, all accessible through an integrated hospital IT system.

The pre‐MDT triage process involved a dedicated meeting held on Wednesday mornings (09:30–11:00), ahead of the formal Friday morning MDT. As set out in the Urology MDT Standard Operating Procedure (SOP), predefined SoC were agreed in advance and used to determine eligibility for streamlining. The MDT coordinator collated all available clinical information and diagnostic reports for review at the pre‐MDT meeting. Cases for which radiology or histopathology reports were unavailable at the time of review were not eligible for streamlining and were referred for full MDT discussion. The pre‐MDT team then assessed each case against the agreed SoC criteria to determine whether it was suitable for protocolised management or required discussion at the full MDT.

Eligibility for streamlining was restricted to predetermined SoCs. For prostate cancer, eligible cases included low‐risk disease (Cambridge Prognostic Group 1) with PIRADS ≤3; nonmetastatic prostate cancer in patients with significant comorbidity precluding radical treatment; metastatic prostate cancer in patients unsuitable for chemotherapy or combination systemic therapy due to comorbidity; postradical prostatectomy cases with negative margins, node‐negative disease and undetectable PSA; and negative prostate biopsies with PIRADS 1–3 lesions.

For bladder cancer, eligible cases included low and intermediate‐risk non‐muscle‐invasive bladder cancer and metastatic bladder cancer in patients with significant comorbidity making them unsuitable for chemotherapy or immunotherapy.

For renal and upper tract cancer, streamlining included patients following nephroureterectomy for noninvasive upper tract urothelial carcinoma (pTa or pT1) with clear surgical margins and no evidence of nodal or metastatic disease, as well as patients following radical or partial nephrectomy for low‐risk renal cell carcinoma (Leibovich score 0–2 for clear cell RCC, or equivalent for nonclear cell RCC) with clear margins and no nodal or metastatic disease.

Cases were excluded from streamlining and referred for full MDT discussion if they fell outside the predefined pathways or were defined within the SoC as requiring full MDT review. This included all testicular and penile cancer cases, any case not meeting the above inclusion criteria, clinically complex or atypical cases, and cases in which radiology or histopathology reports were unavailable at the time of pre‐MDT review.

**FIGURE 1:**
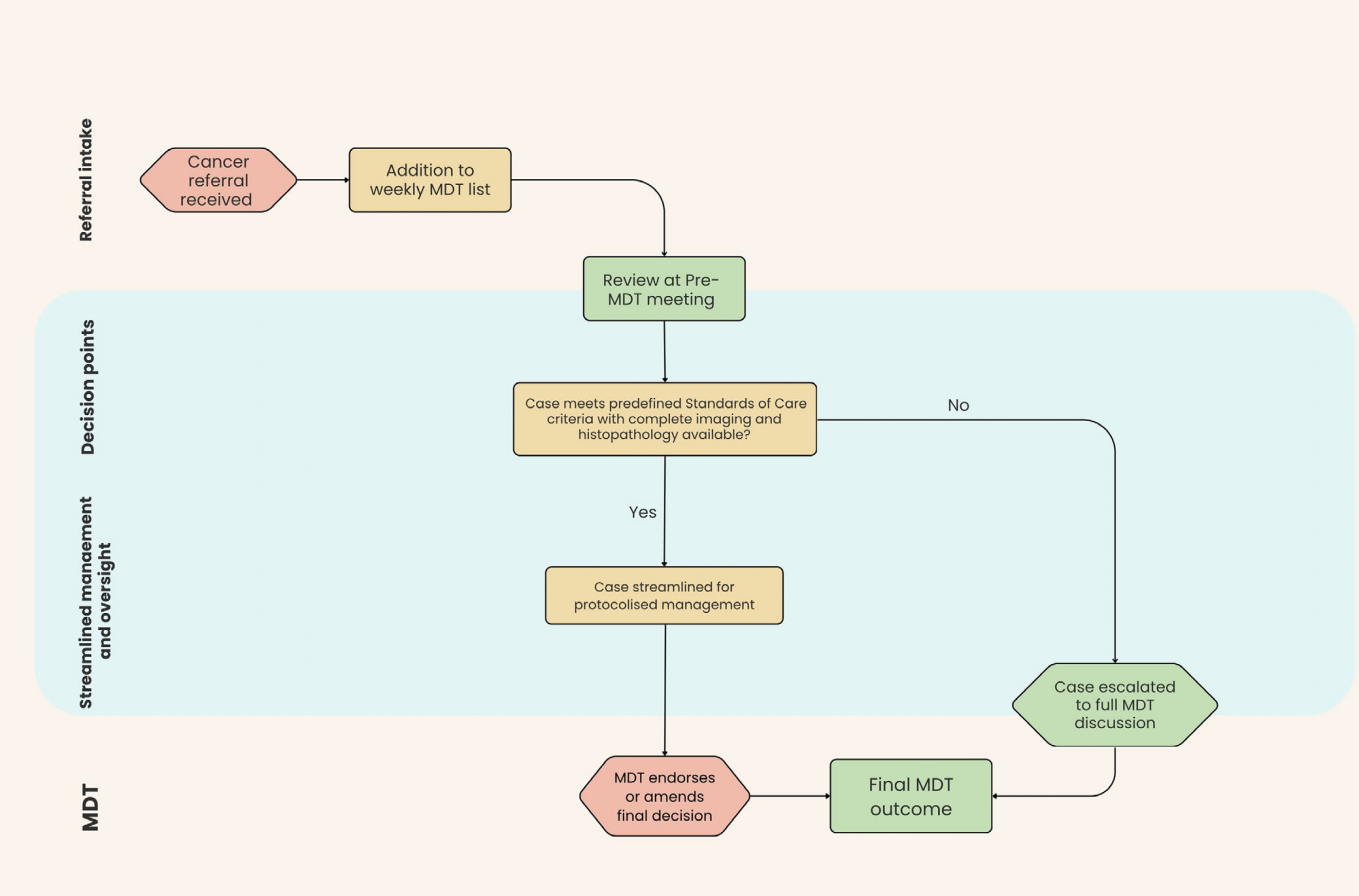
Flow diagram illustrating the pre‐multidisciplinary team (MDT) triage and streamlining pathway.

Streamlined cases were recorded separately as pre‐MDT outcomes and circulated, together with the full MDT case list, to all MDT members in advance of the Friday MDT. Outcomes from pre‐MDT streamlining were not implemented until they had been reviewed and endorsed at the Friday MDT (Figure 1). MDT members were not blinded to pre‐MDT triage decisions, and all cases remained on the MDT agenda with the opportunity for challenge or amendment during full MDT discussion. This early distribution allows team members adequate time to review cases, raise concerns, and prepare for discussion, while also facilitating efficient updating of the cancer registry with provisional outcomes.

This approach protected the team‐based nature of the MDT by ensuring that decisions were not made solely by a single consultant. Instead, all clinicians still had the opportunity to review, challenge, and contribute, keeping the process collaborative and team led.

During the early phase, the team also faced limited engagement when presenting streamlined cases, which occasionally led to passive agreement. To address this, they actively encouraged participation and reinforced that all decisions remained open to full MDT scrutiny.

Data collection was undertaken over 50 weeks, with the programme manager manually recording weekly attendance and collecting MDT data. They entered the data into a structured spreadsheet with two primary tabs:

1 Streamlining Sessions (27 weeks):

This tab records weeks when the team conducted pre‐MDT streamlining. Key variables included the number of cases presented, the number of full‐discussion cases, the number of instances streamlined for approval, the meeting duration, the streamlining duration, the changes in outcome after full MDT, the number of cases not ready for discussion, the meeting finish time, and the date

2 Nonstreamlining Sessions (23 weeks):

This tab captured data from weeks without Pre‐MDT triage, serving as a control for comparison.

The team analysed this data to compare streamlining and nonstreamlining sessions. The analysis focused on the following:

- Comparing total meeting durations to evaluate time efficiency
- Calculating the average time spent on Pre‐MDT cases, which still appeared on the MDT agenda for final input from radiologists and histopathologists (who do not attend Pre‐MDT).
- Identifying any treatment plans modified during the full MDT, despite prior streamlining, to assess the added value of full‐team discussion.

MDT duration data were assessed for normality and were found to be non‐normally distributed. A non‐parametric Mann–Whitney *U* test was therefore used to compare weekly MDT durations between non‐streamlined and streamlined MDT meetings. Statistical analysis was performed using GraphPad Prism, with a two‐tailed test. A p value of <0.05 was considered statistically significant.

To complement the quantitative evaluation, an anonymised survey was distributed to all members of the multidisciplinary team to gather feedback on the streamlining process. The questionnaire aimed to assess perceptions of the pre‐MDT triage model, confidence in the SoC pathways, and the perceived impact on meeting efficiency and clinical decision‐making. The questions were designed by the project lead and finalised by the cancer lead. Responses were anonymised and collected electronically.

The MDT member questionnaire, pre‐MDT triage tasks, and SoCs used for streamlining are provided in Supporting Information.

## 3 RESULTS

During the non‐streamlined period, the urology MDT discussed an average of 32 cases per meeting, with an average meeting duration of 158.3 min and an average discussion time of 4.9 min per case. In the streamlining period, the MDT reviewed an average of 37 cases per meeting, with an average meeting duration of 135 min and average discussion time of 3.6 min. This represented a statistically significant reduction in overall meeting length (*p* < 0.001) (Table 1).

**TABLE 1:** Comparison of discussion times between streamlining and non‐streamlining multidisciplinary team (MDT) sessions.

	Streamlining sessions (27 weeks)	Non streamlining sessions (23 weeks)
Total number of patients	1004	742
Total discussion time (min)	3644	3641
Average time per patient (min)	3.6	4.9
Average weekly MDT discussion duration (min)	135.0	158.3

The duration of streamlining cases was formally recorded in 18 of the 27 weeks. During this period, 733 patients were reviewed, of which 266 cases were streamlined. Streamlined cases accounted for 477 min of discussion, averaging 1.8 min per patient, whereas non‐streamlined cases required 2,123 min, averaging 4.5 min per patient. On average, each meeting included 14 streamlined cases and 24 full‐discussion cases (Table 2).

**TABLE 2:** Comparison of streamlined cases and full multidisciplinary team (MDT) discussions.

	Streamlined cases (weeks 9–27)	Full MDT discussion (weeks 9–27)
Total number of patients	266	467
Average cases per meeting	14	24
Total discussion time (min)	477	2123
Average time per patient (min)	1.8	4.5

**FIGURE 2:**
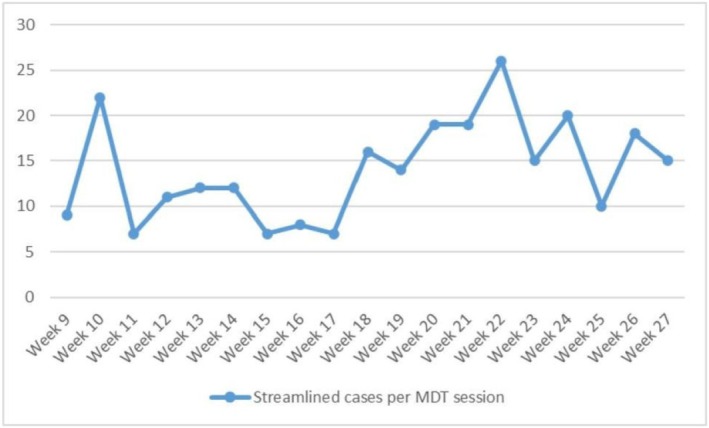
Number of streamlined cases discussed per multidisciplinary team (MDT) session.

During the same period, an average of 4.9 streamlined cases per week had their outcomes amended following full MDT review, representing 35.3% of all streamlined cases. Most of these changes were minor, including clarification of imaging findings or adjustment of surveillance plans. No cases required re‐escalation due to patient safety concerns, and no amendments represented a reversal of the overall treatment plan made from pre‐MDT, indicating that while streamlining improved efficiency, the full MDT continued to play an important role in refining management plans for selected patients. Across the pilot as a whole, 37% of cases were streamlined, reflecting a gradual increase in adoption of the process over time. The number of cases reviewed per streamlining session varied in the early phase of the pilot, ranging from 7 to 22 per session, before stabilising at an average of 15–20 cases per week from week 18 onwards (Figure 2). The median MDT discussion time for streamlined cases was 22 min, aligning with the overall reduction in discussion time per patient observed in the efficiency analysis.

While the streamlining pilot was running, an anonymous survey was distributed to 50 MDT members, with 25 responses received. Most respondents supported the implementation of streamlining, with 79% expressing confidence that patients managed according to SoC pathways would receive appropriate care without requiring full MDT discussion. Regarding time efficiency, 40% reported that the pre‐MDT model significantly reduced meeting time, while 44% considered the time savings to be moderate.

Thirteen respondents offered suggestions for improvement, including integration of AI decision‐support tools, clearer timelines, and increased consultant involvement; 84% of respondents indicated they were willing to engage in further discussions or feedback sessions on the streamlining process. Overall, the survey findings were consistent with the quantitative results, indicating that streamlining had a positive impact on the MDT process while highlighting opportunities for further refinement.

## 4 DISCUSSION

Streamlining MDT processes is increasingly recognised as essential to optimise efficiency, reduce prolonged discussion of straightforward cases, and protect time for complex patients who most benefit from multidisciplinary input. With growing cancer incidence and increasing case complexity, traditional MDT formats are at risk of becoming overloaded, potentially diluting the quality of decision‐making. NHS England's 2020 *Streamlining MDT Meetings Guidance* set the framework for implementing SoC pathways and triaging cases, with the aim of focusing MDT time on complex cases while ensuring oversight for all.^5^

In our pilot, streamlining produced measurable efficiency gains. The average duration of MDT meetings fell from 158 to 135 min (*p* < 0.05). Average discussion time per case decreased from 4.9 to 3.6 min, while streamlined cases required only 1.8 min compared with 4.5 min for non‐streamlined cases. Overall, 37% of cases were managed through the streamlined pathway. Despite this, 94 cases still required modification following full MDT review, underscoring the need for safeguards to prevent misclassification and ensure quality of care.

These findings are comparable with those of Merker et al., who reported that structured premeeting preparation shortened discussion times without compromising decision quality.^1^ Soukup et al. likewise demonstrated that case stratification by complexity can protect time for more detailed deliberation.^6^ Our experience also reflects the principles set out in British Association of Urological Surgeons (BAUS) Oncology guidance, which supports SOC‐based streamlining to improve efficiency while retaining focus on clinically complex patients.^7^

Similar patterns have been described across other oncology specialties. Soukup, Stewart, and Lamb presented evidence‐based approaches to MDT reform that combined efficiency with patient safety by embedding escalation mechanisms for cases not fully aligned with SoC criteria.^8^ Al‐Hammouri et al. reviewed UK practice and found that centres adopting streamlined models generally reported shorter meetings and improved timeliness, though most emphasised the importance of flexibility for atypical or high‐risk cases.^9^

Further comparison with other cancer specialties reinforces our findings. Lung cancer MDTs have reported that efficiency is influenced by case triage, data completeness, and chairing style, with streamlining interventions shown to improve workflow in real‐world evaluations.^10^ Similarly, systematic reviews of colorectal MDTs highlight the wide variation in how cases are prepared and presented, with streamlining via SoC pathways recommended to reduce duplication and focus resources on complex patients.^11^, ^12^ In upper gastrointestinal oncology, implementation of timed diagnostic pathways and regional SoC standards has demonstrated that streamlining accelerates decision‐making and reduces bottlenecks.^13^, ^14^ In gynaecological oncology, pathway reforms have similarly emphasised pre‐MDT triage and standardisation to ensure that only cases requiring multidisciplinary debate consume full meeting time.^15^ These cross‐specialty examples reinforce that our results are not isolated, but part of a broader trend across UK cancer services.

Our post‐pilot questionnaire results echo the literature in demonstrating strong clinician support. The majority of respondents (79%) were confident that SoC cases receive appropriate care without full MDT discussion, and most perceived significant (40%) or moderate (44%) time savings. Notably, 71% reported improved quality of discussions overall, while 84% were willing to remain engaged with refinement of the process. Support for streamlining among MDT members increased as the pilot progressed, although a formal follow‐up survey was not conducted. These findings are consistent with reports in the wider literature where staff buy‐in, clarity of pathways, and communication are identified as critical enablers of sustainable streamlining.^16^ These findings mirror national surveys in which MDT members expressed strong support for reform and selective case discussion to safeguard meeting quality.^17^

The evidence on streamlining cancer multidisciplinary team (MDT) meetings in Urology shows consistent support for reform tempered by concerns over governance, safety, and clinical standards. Early surveys confirmed that MDTs are valued but become inefficient when burdened by large numbers of low‐risk or routine cases. Prioritising complex patients and adopting pre‐approved pathways were advanced as practical solutions, though clinicians warned against fragmenting discussions.^18^ National analyses echoed these tensions: broad consensus on structured triage and protocol‐driven reform set against anxieties about documentation quality and potential safety risks.^16^ Mixed‐methods work has since shown that reducing routine case reviews can deepen deliberation for complex patients, but only when standards, defined pathways, and stakeholder agreement are in place.^19^

Digital and AI‐driven platforms have introduced new possibilities. A digital MDT platform improved workflow efficiency, documentation, and surgical planning, yet adoption is limited by training and technological capacity.^20^ The PROSAIC‐DS AI triage system allowed one third of prostate cancer cases to bypass full discussion without compromising concordance, though the authors caution that governance, validation, and oversight are indispensable.^21^ Systematic reviews continue to affirm MDTs' benefits for multidisciplinary input, guideline adherence, and potential outcome gains, but stress the low quality of evidence and lack of controlled trials.^22^, ^23^

Our pilot points to a recalibrated model: objective triage, protocol‐driven handling of routine cases, and concentrated debate on complex decisions. Yet every step toward streamlining must be anchored in strict governance, detailed documentation, and continuous monitoring to protect safety and accountability.

While the benefits of streamlining were clear, several challenges remain. First, the process currently relies on a single consultant urologist for pre‐MDT triage, meaning the system is vulnerable to disruption if that individual is unavailable due to leave or other commitments. Second, the success of streamlining is highly dependent on the timely availability of histopathology and radiology reports. If they are delayed, cases cannot safely be triaged to SoC pathways and must instead be escalated for full MDT discussion.

Although SoC‐based triage can increase efficiency, continued oversight within the full MDT remains essential as a safeguard to ensure that atypical features, comorbidities, or patient preferences are not overlooked. Future models of pre‐MDT triage may also benefit from the inclusion of a radiologist and/or pathologist, allowing focused diagnostic review prior to the formal MDT; however, implementation would require careful consideration of workforce capacity. In addition, future iterations of MDT streamlining may benefit from the formal allocation of protected time for MDT members to review circulated case lists in advance, although this would require incorporation into individual job plans. These limitations highlight the importance of robust safety‐netting, cross‐cover arrangements, and ongoing review to ensure sustainability of the streamlining model.

## 5 CONCLUSION

To our knowledge, this is the first study to evaluate the implementation and impact of a pre‐MDT streamlining model specifically within a Urology cancer service. The streamlining interventions implemented by the Urology MDT at Dartford and Gravesham NHS Trust demonstrated meaningful improvements in meeting efficiency and clinician confidence in protocolised care pathways. While there remains scope for further refinement and broader application, these results support the viability of structured pre‐MDT triage and SoC‐based decision‐making. The approach provides a model that other NHS trusts can adopt to enhance the quality and timeliness of multidisciplinary cancer care. Building on this foundation, we are now exploring the integration of AI tools to support clinical decision‐making in the pre‐MDT setting. A follow‐up study is underway to evaluate the potential of AI‐assisted triage in further improving MDT performance and patient outcomes.

## AUTHOR CONTRIBUTIONS

Sanjeev Madaan conceptualised and supervised the study, contributed to the creation of the standard operating procedure (SOP) for streamlining, and assisted with writing and editing. Srijit Banerjee helped create the streamlining SOP. Adeyinka Pratt collected the data and performed the initial analysis and draft. Hossein Arang, Muhammed Rashim Parappan, and Mutahhar Nabeel Syed contributed to data analysis and manuscript revision. Jayasimha Abbaraju assisted with data collection. All authors reviewed and approved the final version of the manuscript.

## CONFLICT OF INTEREST STATEMENT

The authors declare no conflicts of interest related to this work.

## Supporting information


**Data S1.** Supporting Information.
