# Supplementary material for: Streamlining urology cancer multidisciplinary team meetings: Implementation and outcomes
Source: BJUI Compass. 2026 Feb 5;7(2):e70167. doi: 10.1002/bco2.70167 (PMC12875837; doi:10.1002/bco2.70167)
Supplement: Supplementary file 1 — Data S1. Supporting Information. [file BCO2-7-e70167-s001.docx]

**Section A:** Multidisciplinary Team (MDT) Streamlining Survey Questionnaire


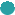

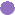
Responses Overview Active


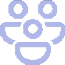


Responses

25


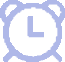


Average Time

05:48


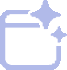


Duration

99 Days

1.
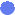

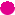

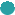

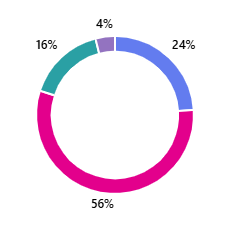
Do you agree with the principle of categorising patients into two groups (those requiring full discussion vs. those managed by the Standard of Care)?

| Strongly Agree | 6 |
| --- | --- |
| Agree | 14 |
| Neutral | 4 |
| Disagree | 1 |
| Strongly disagree | 0 |

1.
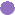

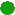

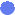

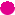

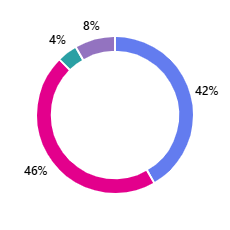
How familiar are you with the Urology MDT streamlining process?

| Very Familiar | 10 |
| --- | --- |
| Familiar | 11 |
| Relatively Familiar | 1 |
| Not Familiar | 2 |

1.
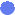

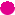

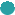

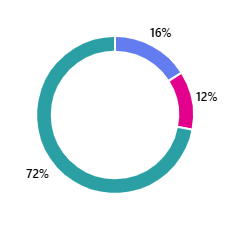

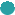

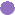
How would you prefer cases with a suggested treatment plan from the Wednesday meeting to be handled during the Friday MDT? Please Tick your preferred approach:

| No discussion and not on the list | 4 |
| --- | --- |
| No discussion and on the list | 3 |
| Minimal discussion and on the list | 18 |
| Full discussion and disagreement with streamlining | 0 |

1.
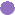

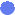

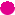

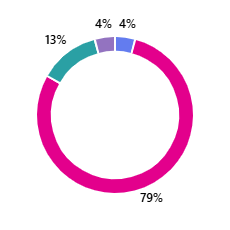
Do you feel confident that patients whose needs are met by the Standard of Care (SoC) will receive appropriate care without being discussed at the full MDT?

| Very Confident | 1 |
| --- | --- |
| Confident | 19 |
| Neutral | 3 |
| Not so confident | 1 |

1.
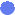

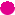

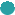

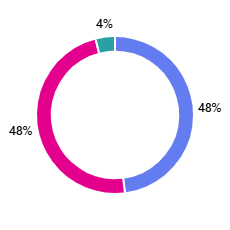
How effective do you believe this streamlining process will be in improving the efficiency of MDT discussions?

| Very effective | 12 |
| --- | --- |
| Somewhat effective | 12 |
| Neither effective nor ineffective | 1 |
| Somewhat ineffective | 0 |
| Very ineffective | 0 |

1.
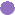

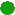
Do you anticipate any challenges or risks with the streamlined process, such as incorrect patient categorisation? Please state in the box below

# 20

Responses

Latest Responses

"No"

"No"

"No"

*. . .*

4 respondents (20%) answered patients for this question.

incorrect reportsradiology and pathology information list/MDT MDT discussiondiscussion at MDM

patient availablePatients cases **patientstimes** reports patient assessment

MDT list minimal discussion

great amount of patients

list

consultant cover

streamlined patients

Current challenges

MDT meeting

1.
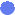

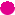

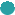

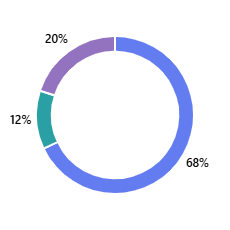

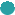

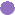
How do you think the streamlining process will impact patient outcomes and the quality of care?

| Positive Impact | 17 |
| --- | --- |
| Negative impact | 0 |
| No impact | 3 |
| Unsure | 5 |

1.
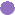

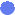

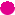

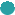

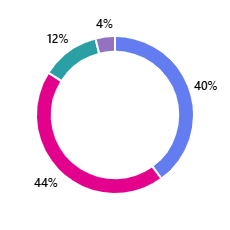
How much time do you estimate has been saved in MDT discussions due to the streamlining process?

| Significant time saved (more than 40%) | 10 |
| --- | --- |
| Moderate time saved (10% - 40%) | 11 |
| Minimal time saved (less than 10%) | 3 |
| No time saved | 1 |

1.
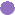

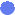

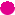

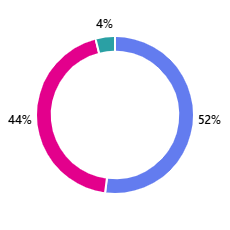
Do you feel that the time saved has contributed to discussions that are more effective during MDTs?

| Yes, greatly | 13 |
| --- | --- |
| Yes, Relatively | 11 |
| No, it hasn’t made a difference | 1 |
| No, it has had a negative impact | 0 |

1.
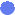

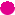

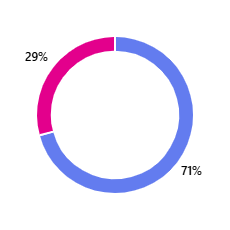
How has the reduction in discussion time for some patients affected the overall quality of MDT meetings?

| Quality has improved | 17 |
| --- | --- |
| Quality has remained the same | 7 |
| Quality has decreased | 0 |

1.
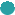
Do you think the streamlined process has improved the timeliness of decision-making during MDTs?


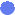

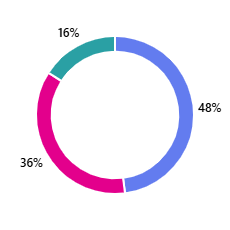
Yes, decision-making has been faster and more

efficient 12


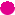
 Relatively, but there’s room for improvement 9


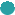
 No, decision-making speed has not changed 4


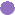
 No, decision-making has become slower 0

1. What additional resources, tools, or support would help the team implement this streamlined process successfully?

# 13

Responses

Latest Responses

"AI"

"Better Decision"

*. . .*

2 respondents (15%) answered option of AI for this question.

Path and radiology

**consultants** pre streamline team

histology and radiology

urology team **support**

streamlined process patients being put on mdm

presence of another urologist

**option of AI**

decision makingtimely

**better decision** helpful

MDT Team **reports**

open mind

**radiology** MRI meeting

Consistency of consultants

1. Do you have any suggestions for improving the Urology MDT streamlining process?

# 13

Responses

Latest Responses

"Regular weekly meetings"

*. . .*

3 respondents (23%) answered times for this question.

understanding and support

Radiology and Histology

no consultant consultants streamlining process

consultants rota

MDT team

**times**

streamline MDT

going forward

**plan**

consultant is not

supporting

monthly caths

Friday afternoon

MDT clinic

meetings Radiology

clinician and CNS

reports are accurate

1.
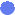

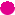

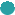

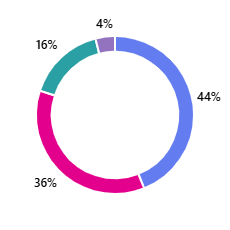

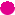

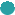
How satisfied are you with the communication regarding the MDT streamlining pilot?

| Very satisfied | 11 |
| --- | --- |
| Somewhat satisfied | 9 |
| Neither satisfied nor dissatisfied | 4 |
| Somewhat dissatisfied | 1 |
| Very dissatisfied | 0 |

1.
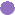

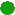

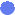

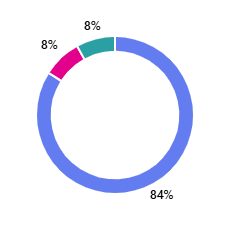
Would you be willing to participate in further discussions or feedback sessions regarding the streamlining process?

| Yes | 21 |
| --- | --- |
| No | 2 |
| Maybe | 2 |

**Section B:** Tasks of the Pre-Multidisciplinary Team (MDT)

The pre-MDT team undertakes structured case review and documentation in preparation for formal MDT discussion. Key tasks include:

- Ensuring that all available clinical information and diagnostic reports are available for structured review and documentation at the pre-MDT meeting on Wednesday morning.
- Reviewing cases against predefined Standards of Care, as set out in the Urology MDT Standard Operating Procedure, to determine eligibility for MDT streamlining.
- Confirming the availability of radiology and histopathology reports for each case.
- Documenting streamlined cases separately as pre-MDT outcomes.
- Circulating the MDT case list and pre-MDT outcomes to all MDT members ahead of the Friday MDT meeting.
- Ensuring that outcomes from pre-MDT streamlining are not implemented until reviewed and endorsed at the formal Friday morning MDT meeting.

Section C: Standards of Care (SoC) for MDT Streamlining

# The following Standards of Care are reproduced from the local Urology MDT Standard Operating Procedure for MDT streamlining. Streamlining criteria for discussion at the pre-MDT meeting are listed below.

# **Prostate cancer streamlining**

| Risk Group | Standard of Care Guidelines |
| --- | --- |
| - CPG1 with PIRAD ≤ 3 | **These patients can be streamlined to SoC without being discussed in MDM.**  **SoC** recommends active surveillance as first choice.  Consideration for radical therapy (surgery/radiation/brachytherapy) may arise  if a patient declines active surveillance or  if the patient's life expectancy exceeds 20 years, although the advantages of surveillance may be more significant for younger men.  Or if histology indicates multifocal prostate cancer |
| - CPG 2-5 *-* suitable for radical treatment | **Discuss all cases at MDM** |
| - Post – RALP | **Discuss all cases at MDM** if margin +ve or N1 or PSA detectable |
| - Non-metastatic prostate cancer cases with marked comorbidity making ineligible for radical therapy | **These patients can be streamlined to SoC without being discussed in MDM**    **SoC** recommends watchful waiting for these patients |
| - Metastatic prostate cancer cases- where they are suitable for further oncological treatment i.e. PS 0 or 1 | **Discuss all cases at MDM** |
| - Metastatic prostate cancer with marked co-morbidity making patient not suitable for chemotherapy or combination therapy | **These patients can be streamlined to SoC without being discussed in MDT**  **SoC** recommends ADT monotherapy for these patients.  In case of symptoms, contemplate a referral to oncology. |
| - All patients who had been previously treated for prostate cancer and have failed first line therapy and fit for salvage treatment. - MRI PIRADS 4-5 & negative biopsy | **Discuss all cases at MDM**  **Discuss all cases at MDM** |
| - Negative biopsies PIRAD 1-3 | **SoC recommends discharge with PSA threshold for re-referral** |

**Bladder Cancer Streamlining**

| Risk Group | Standard of Care |
| --- | --- |
| - 1. Low risk NMIBC - Solitary pTaG1 with a diameter of less than 3 cm - Solitary pTaG2 (low grade) with a diameter of less than 3 cm - Any papillary urothelial neoplasm of low malignant potential (PUNLMP) | **These patients can be streamlined to SoC without being discussed in MDM**  **SoC** recommends cystoscopic surveillance in accordance with NICE or EAU guidelines. |
| - 1. Intermediate risk NMIBC - Solitary pTaG1 with a diameter of more than 3 cm - multifocal pTaG1 - Solitary pTaG2 (low grade) with a diameter of more than 3 cm - Multifocal pTaG2 (low grade) - pTaG2 (high grade) - Any pTaG2 (grade not further specified) – - Any low-risk non-muscle-invasive bladder cancer recurring within 12 months of last tumour occurrence | **These patients can be streamlined to SoC without being discussed in MDM**  **SoC** recommends intravesical Mitomycin C treatment once a week for 6 weeks and regular cystoscopic surveillance in line with NICE/EAU guidelines. |
| 1. High risk NMIBC  - pTaG3 - pT1G2 - pT1G3 - pTis (Cis) - Aggressive variants of urothelial carcinoma, for example micropapillary or nested variants - All non-Urothelial cancers such as Squamous Cell Carcinoma, Adenocarcinoma, Small Cell Carcinoma, Unusual Histological Subtypes (Lymphoma etc) | **Discuss all cases at MDM** |
| 1. Muscle invasive bladder cancer | **Discuss all cases at MDM** |
| 1. Metastatic bladder cancer suitable for chemotherapy | **Discuss all cases at MDM** |
| 1. Metastatic bladder cancer with marked comorbidity making not suitable for chemotherapy or immunotherapy | **These patients can be streamlined to SoC without being discussed in MDM**  **SoC** recommends Symptomatic Control Only +/- Referral to Community Palliative Care Team |

# **Renal and Upper Tract Cancer Streamlining**

| Risk Group | Standard of Care |
| --- | --- |
| - 1. All patients referred with a suspected new diagnosis of renal cell cancer or upper tract urothelial cancer | **Discuss all cases at SMDM** |
| - 1. Patients after nephroureterectomy with non-invasive urothelial cancer on final histology - pTa or PT1 - Clear surgical margins - No evidence of nodal or metastatic disease (normal pre-operative staging) | **These patients can be streamlined to SOC without being discussed in MDM**  **SOC** recommends Flexible cystoscopy in 3 months |
| - 1. Patients after radical or partial nephrectomy with low-risk renal cell cancer on final histology - Leibovich score 0-2 for clear cell RCC (or equivalent for non- clear cell RCC) - Clear surgical margins - No evidence of nodal or metastatic disease   ( normal pre-operative staging) | **These patients can be streamlined to SOC without being discussed in MDM**  **SOC Recommends CT TAP at 6, 18 and 36 months and then at year 5** |
| - 1. All patients after nephroureterectomy with invasive urothelial cancer (pT2-pT4) on histology | **Discuss all cases at MDM** |
| - 1. All patients after radical or partial nephrectomy   with intermediate or high-risk renal cell cancer  or histology   - - - pT2 N0 M0 disease with either Fuhrman grade 4 or sarcomatoid histology (intermediate-high risk)     - pT3 N0 M0 with any histological grade (intermediate-high risk) or - pT4 N0 M0 with any histological grade (high risk)     - any pT N1 M0 with any histological grade (high risk) or - any pT any N M1 with no evidence of disease after complete resection of both loco-regional disease and all metastatic lesion(s) | **Discuss all cases at MDM and consider adjuvant treatment** |
| - 1. All patients with small renal masses on active surveillance who are being considered for a change in management to active treatment | **Discuss all cases at MDM** |
| 1. All patients with metastatic renal cell cancer or metastatic upper tract urothelial cancer | **Discuss all cases at MDM** |
| 1. All patients with recurrent renal cell cancer or recurrent upper tract urothelial cancer | **Discuss all cases at MDM** |

# **Testis Cancer Streamlining**

- All testis cases to be discussed in MDM.

# **Penis Cancer Streamlining**

- All need to discussed at MDM and referred to supra- regional centre.
